# Silencing Osteopontin Expression Inhibits Proliferation, Invasion and Induce Altered Protein Expression in Melanoma Cells

**DOI:** 10.3389/pore.2021.581395

**Published:** 2021-03-05

**Authors:** Tímea Kiss, Krisztina Jámbor, Viktória Koroknai, István Szász, Helga Bárdos, Attila Mokánszki, Róza Ádány, Margit Balázs

**Affiliations:** ^1^ Department of Public Health and Epidemiology, Faculty of Medicine, University of Debrecen, Debrecen, Hungary; ^2^ Doctoral School of Health Sciences, University of Debrecen, Debrecen, Hungary; ^3^ MTA-DE Public Health Research Group, University of Debrecen, Debrecen, Hungary; ^4^ Department of Pathology, Faculty of Medicine, University of Debrecen, Debrecen, Hungary

**Keywords:** osteopontin expression, osteopontin knockdown, protein expression profile, melanoma progression, invasion, proliferation

## Abstract

Osteopontin (OPN) is a multifunctional phosphoprotein that is expressed in different types of cancers, including melanoma. OPN overexpression is associated with tumor progression and metastasis formation; however, the role of OPN in cell invasion and metastasis formation is not completely understood. In this study we aimed to define OPN expression in melanoma tissues and cell lines and investigate the effect of OPN expression on cell proliferation and invasion after inhibiting OPN expression with small interfering RNA (siRNA). OPN gene expression was determined by qRT-PCR, while protein expression was examined using a Proteome Profiler Oncology Array. siRNA-mediated OPN knockdown led to decreased OPN expression in melanoma cell lines, which was associated with decreased cell proliferation and invasion. Proteome profile analysis revealed significantly different protein expression between the original and transfected cell lines. The altered expression of the differently expressed proteins was validated at the mRNA level. Furthermore, OPN-specific siRNA was able to reduce OPN expression and inhibit the invasiveness of melanoma cells. Our results revealed for the first time that silencing the OPN gene influences proliferation and invasion of melanoma cells by effecting EGFR, tenascin C, survivin, galectin-3 and enolase 2 expression. To predict protein-protein interactions along with putative pathways we used STRING analysis for the differentially expressed proteins. These proteins formed multiple clusters, including extracellular matrix organization, regulation of angiogenesis, cell death and cell migration, PI3K-Akt, MAPK and focal adhesion signaling pathways. Taken together these data suggest that OPN might be an ideal target for drug development and therapies.

## Introduction

Cutaneous melanoma is one of the most invasive and metastatic human cancers and accounts for the majority of skin cancer deaths despite comprising less than 5% of all cutaneous malignancies [1, 2]. Although, local excision of early-stage primary melanoma offers the best chance of cure, recent advances in molecular genetics and genomics have revolutionized the management and treatment of late-stage and metastatic melanomas, leading to significant improvements in clinical outcomes [3, 4]. Recently, numerous studies have suggested that osteopontin (OPN) plays a crucial role in cancer progression in different malignancies, including malignant melanoma [5–8]. Previous reports indicated that upregulated OPN expression was correlated with tumor cell migration, invasion, progression and metastasis formation [9–12]. Tumor metastasis is a multistep process that includes tumor invasion and intravasation into the vessels. This is followed by the survival of tumor cells in the circulatory system and their extravasation into distant tissues, where they may be able to proliferate [13–15]. OPN enhances the survival of cancer cells through its interaction with CD44 isoforms (CD44v) on the cell surface [16, 17], and it can promote tumor metastasis by interacting with different types of integrins (αvβ1, αvβ3, αvβ5, and α5β1) [18, 19]. However, the molecular mechanism through which OPN promotes the invasion and metastasis formation of cutaneous melanoma remains unclear. Several studies indicate that OPN could be a specific target for cancer therapy [6, 8, 20]. Currently, the broadly accepted OPN expression inhibition by RNA interference (RNAi) seems to be a promising strategy for cancer treatment [21 – 24]. In this study, our aim was to determine OPN expression at both the mRNA and protein levels in melanoma cell lines and OPN gene expression in primary and metastatic tissues. OPN expression knockdown was shown by several authors to have anti-metastatic and anti-tumorigenic effects [23, 25]. We also aimed to inhibit OPN expression by RNAi in selected melanoma cell lines, which are characterized by high OPN expression, and examine the effect of transfection on the cells. The role of OPN in tumorigenesis is complex and likely has different effects in different tumors. To better understand the molecular events of OPN responsible for malignancy, we determined the protein expression patterns of original and transfected cell line pairs (primary tumor and metastasis-derived melanoma cell lines originating from the same patients) using proteome analysis.

## Materials and Methods

### Melanoma Cell Lines

Melanoma cell lines were obtained from the Coriell Institute for Medical Research (Camden, NJ, United States). Cell lines were cultured in RPMI 1640 medium (Lonza Group Ltd., Basel, Switzerland) and supplemented with 10% fetal bovine serum (Gibco, CA, United States) at 37°C in an atmosphere containing 5% CO2. The clinical-pathological characteristics of the cell lines are summarized in Table 1.

**TABLE 1:** Characteristics of human melanoma cell lines.

Cell line	Origin^a^	Growth phase^b^	Histologic type^c^	BRAF mutation status	NRAS mutation status
WM35	Primary	RGP/VGP	SSM	V600E	wt
WM793B	Primary	RGP/VGP	SSM	V600E	wt
WM3211	Primary	RGP/VGP	SSM	wt^d^	wt
WM902B	Primary	VGP	SSM	V600E	wt
M35/01	Primary	VGP	SSM	V600E	wt
WM1361	Primary	VGP	SSM	wt	Q61L
WM1366	Primary	VGP	SSM	wt	Q61L
HT199	Primary	RGP	NM	V600E	wt
WM39	Primary	VGP	NM	V600E	wt
WM3248	Primary	VGP	unknown	V600E	wt
WM278^5^ ^e^	Primary	VGP	NM	V600E	wt
WM1617^5^ ^f^	Metastasis	—	—	V600E	wt
WM983A^6^ ^e^	Primary	VGP	NM	V600E	wt
WM983B^6^ ^f^	Metastasis	—	—	V600E	wt
SK-MEL-28	Metastasis	—	—	V600E	wt
A2058	Metastasis	—	—	V600E	wt
HT168-M1	Metastasis	—	—	V600E	wt
M24	Metastasis	—	—	wt	Q61R
M24 met	Metastasis	—	—	wt	Q61R
Melur	Metastasis	—	—	wt	wt

^a^ Origin of cell lines.

^b^ RGP: radial growth phase, VGP: vertical growth phase.

^c^ SSM: superficial spreading melanoma, NM: nodular melanoma.

^d^ wt: wild-type.

^e^ Primary tumor derived cell line with metastatic pair from the same patient.

^f^ Metastatic pair of primary tumor derived cell line.

### Melanoma Tissue Samples

Melanoma tissues were obtained from the Department of Dermatology, University of Debrecen, Hungary. This study was approved by the Regional and Institutional Ethics Committee of the University of Debrecen, Hungary (DE RKEB/IKEB: 4820-2017) and was carried out according to all relevant regulations. Written informed consents obtained from the patients. Lesions were diagnosed on the basis of formalin-fixed paraffin-embedded tissue sections stained with hematoxylin–eosin. A total of 34 primary and 12 metastatic melanoma samples were used for qRT-PCR. The clinical-pathological parameters of the tumors are summarized in Supplementary Table S1.

### RNA Extraction and qRT-PCR Analysis

RNeasy Plus Mini Kit (Qiagen GmbH, Hilden, Germany) was used to isolate total RNA according to the manufacturer’s protocol. The concentrations of the RNA samples were determined using a NanoDrop ND-1000 UV-Vis spectrophotometer (NanoDrop Technologies, Wilmington, DE, United States). Reverse transcription (RT) was performed on total RNA (600 ng) using a High-Capacity cDNA Reverse Transcription Kit according to the manufacturer’s protocol (Life Technologies Corporation, Carlsbad, California, United States). To perform qPCR reactions, SYBR premix Ex Taq (Takara Holding Inc., Kyoto, Japan) master mix was used. Raw PCR data were analyzed using the Livak method (2-ΔΔCt) with glyceraldehyde-3-phosphate dehydrogenase (GAPDH) as an internal control gene and cultured melanocyte or pooled nevi (*n* = 8) as the calibrator sample [26].

### siRNA Experiments

siRNA directed against human secreted phosphoprotein 1 (SPP1; OPN-targeting siRNA: ID: SI02757615) and AllStars Negative Control siRNA (NC-siRNA ID: 1027281) were purchased from Qiagen GmbH (Hilden, Germany). The sequences of the OPN (SSP1) specific siRNA are: sense: 5’-GGC​UGA​UUC​UGG​AAG​UUC​UTT-3’; antisense: 5’-AGA​ACU​UCC​AGA​AUC​AGC​CTG-3’. The sequence-specific highly functional OPN silencing siRNA was validated on OPN gene expression by the manufacturer [27], the potential off-target mRNA was none, the siRNA has a high homology (https://geneglobe.qiagen.com/product-groups/flexitube-sirna). The specificity of the sequence was also checked in the BlastN database. In agreement with published data, to avoid the off-target effects (non-specific binding) we applied the lowest effective concentration (5 nM) of the siRNA during our experiments [28].

One day before transfection, cells were seeded in 24-well plates at a density of 5 × 10^4^ cells per well and cultured in 500 μL of growth medium without antibiotics to 30–50% confluence. Small interfering RNA (siRNA) duplex-Lipofectamine 2000 transfection reagent (Invitrogen, Life Technologies, Carlsbad, CA, United States) complexes (a final volume of 100 μL and a final siRNA concentration of 5 nM) were added to each well according to the manufacturer’s protocol. After 3 h of incubation, the medium was replaced with fresh medium. The cells were harvested 48 h after transfection for analysis. Gene silencing efficacy was assessed by qRT-PCR. All the transfections were repeated three times independently.

### Cell Proliferation Assay

At 48 h after transfection, WST-1 Cell Proliferation Reagent (Roche Magyarország Kft., Budaörs, Hungary) was used according to the manufacturer’s instructions to measure cell proliferation as an indicator of undesirable RNAi activity. Absorbance was measured at 450 nm using a NanoDrop ND-1000 UV-Vis Spectrophotometer (NanoDrop Technologies, Wilmington, DE, United States). The reference absorbance was set at 700 nm. Cell proliferation was measured and compared with that of control cells. Each experiment was carried out independently and repeated at least three times.

### *In vitro* Invasion Assay

The invasive potential of the melanoma cell lines was analyzed using BD Biocoat Matrigel invasion chambers (pore size: 8 μm, 24-well; BD Biosciences, Bedford, Massachusetts, United States) as described by Koroknai et al. [29]. The upper chamber of the insert was filled with 500 μL of cell suspension in serum-free media (5 × 10^4^ cells/well). Medium containing 10% FBS was applied to the lower chamber as a chemoattractant. Tumor cells were incubated for 24 h at 37°C. After non-invading cells were removed with a cotton swab, the invading cells in the lower layer were fixed with methanol and stained with hematoxylin–eosin. The average number of invaded cells was counted using a light microscope in seven different visual fields at ×200 magnification. The data are presented as the mean ± SD of three independent experiments.

### Protein Expression Analysis

Cells were cultured to approximately 80% confluence in T25 flasks and gently washed twice with 10 ml of ice-cold PBS. After adding 1 ml of RIPA Lysis and Extraction Buffer (Thermo Fisher Scientific Inc. Waltham, Massachusetts, United States) containing 20 µL of Halt™ Protease and Phosphatase Inhibitor Cocktail (Thermo Fisher Scientific Inc. Waltham, Massachusetts, United States) to each flask, a cell scraper was used to scrape the cells. Then, the cell lysates were transferred to a new Eppendorf tube, incubated on a rocking shaker for 30 min at 4°C, and centrifuged at 13.000 rpm for 30 min at 4°C. The supernatant was collected (avoiding the pellet) in new microtubes. The protein concentration was determined with a Quick Start™ Bradford Protein Assay (Bio-Rad Hungary Ltd. Budapest, Hungary) performed according to the manufacturer’s protocol.

Protein expression was investigated using a Proteome Profiler Human XL Oncology Array Kit (R&D Systems, Abingdon, United Kingdom) according to the instructions of the supplier. Cell lysates were obtained, and 84 different proteins were analyzed in duplicate. Cell lysates (200 µg) were incubated with each array overnight at 4°C on a rocking platform shaker. On the following day, the cell lysates were removed, and the membranes were washed 3 times with wash buffer. After incubating the arrays with a detection antibody cocktail for 1 h at room temperature on a rocking platform shaker, the membranes were washed 3 times with wash buffer. Then, 2 ml of streptavidin–HRP mix was added to each membrane for a 30 min incubation, followed by three washes. The labeled protein spots were visualized using Chemi Reagent Mix. Array spots were analyzed with ImageJ Lab 1.51 Software and normalized to positive control signal intensities (1.51a, NIH, Bethesda, Maryland, United States) and evaluated by subtracting the background. The intensity of the reference spots was considered as 100%.

The protein–protein functional associations investigated by STRING (Search Tool for the Retrieval of Interacting Genes) database version 11.0 (http://string-db.org) which is web-based tool that collected associations between proteins from multiple sources [30].

### Statistical Analysis

SPSS (Statistical Package for the Social Sciences) 22.0 software (SPSS Inc., Chicago, IL, United States) was used for statistical analysis. All experiments were carried out at least three times, and the data are presented as the means (±standard deviation). The Kruskal-Wallis test was used to determine the significant differences among OPN gene expression in melanoma subgroups (SSM, NM and melanoma metastasis). A two-sided Mann-Whitney-Wilcoxon exact test was used to reveal significant differences between primary and metastatic melanoma cell lines. Student’s t-test was performed for the statistical analysis of the experimental siRNA data. A *p* value ≤ 0.05 was considered statistically significant.

## Results

### OPN Gene Expression Patterns in Melanoma Cell Lines and Primary and Metastatic Melanoma Tissues

**FIGURE 1:**
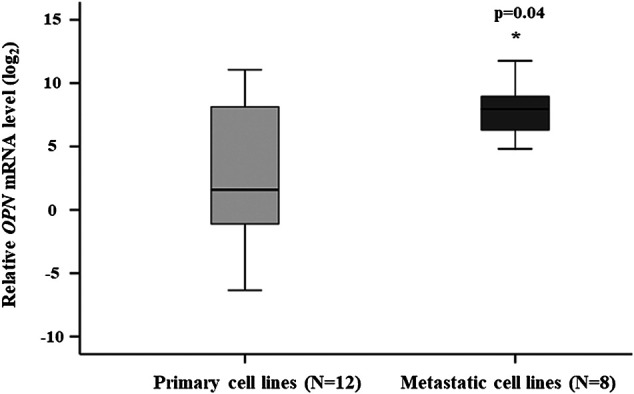
Comparison of the relative OPN mRNA expression in the primary and metastatic cell lines. The data are presented as the mean ± SD of the primary (*n* = 12) and metastatic (*n* = 8) cell lines (three replicates/sample). Significant differences (*p* ≤ 0.05; Mann-Whitney test) are indicated by asterisks. Cell lines characteristics are summarized in Table 1.

OPN expression was determined by qRT-PCR in melanoma cell lines (*n* = 20). The cell lines originated from primary melanomas (*n* = 12) and melanoma metastases (*n* = 8). The relative OPN mRNA expression was significantly enhanced in the metastatic cell lines (*p* = 0.04) (Figure 1). The OPN expression in the *BRAF* ^*V600E*^ mutant cell lines (*n* = 14) were significantly higher compared to cell lines with wild-type BRAF (*n* = 6) (Supplementary Figure S1).

We also determined the OPN gene expression level in primary and metastatic tumor tissues by qRT-PCR. The clinical parameters of melanoma patients are summarized in Supplementary Table S1. The highest relative expression was found in a melanoma metastasis (84.5). Of the 12 metastatic samples eight had the OPN relative gene expression level ranged between 13.1 and 84.5. Three primary tumors of the aggressive nodular subtypes (*n* = 10) also exhibited high relative gene expression level (range: 17.6–61.8), tissues of the less aggressive SSM subtypes (*n* = 24) showed a more uniform pattern with low OPN gene expression (range: 0.1–9.14). Significant differences were seen between the subgroups (*p* = 0.0005; Kruskal-Wallis test) (Supplementary Figure S2).

### Effect of RNAi on OPN Expression

**FIGURE 2:**
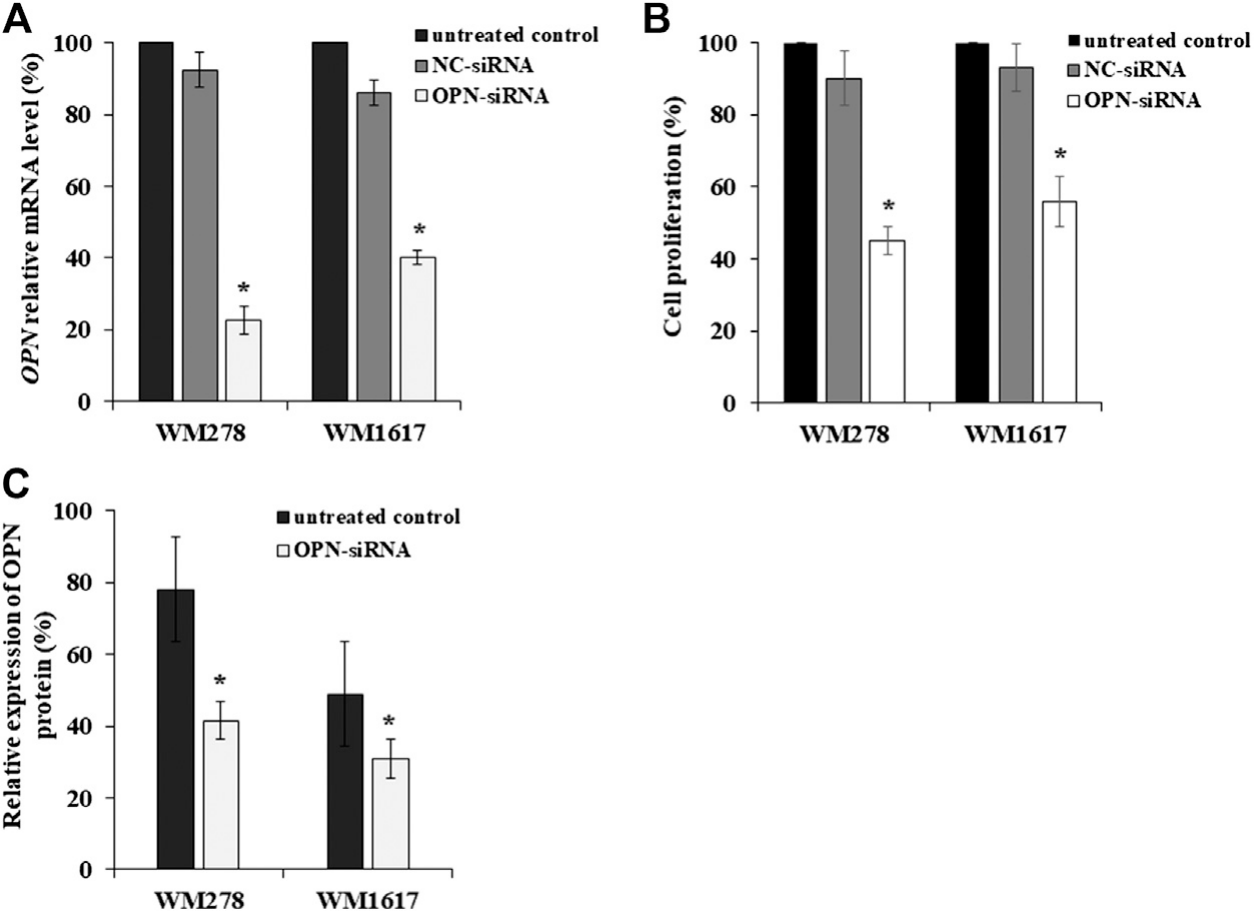
Inhibition OPN expression in melanoma cell lines by OPN-siRNA. **(A)** Relative expression of OPN-mRNA after 5 nM of AllStars Negative Control siRNA (NC-siRNA) and OPN-siRNA transfection. Significantly reduced OPN mRNA expression was observed in all OPN-siRNA transfected cell lines compared to control cells (*p* ≤ 0.05; Mann-Whitney test). **(B)** Cell proliferation of untreated, NC-siRNA and OPN-siRNA treated cell lines. Significant decrease of cell proliferation was detected in OPN-siRNA silenced melanoma cells compared to the NC-siRNA transfected and untreated control cells (*p* ≤ 0.005; Student’s t-test). **(C)** OPN protein expression in untreated and OPN-siRNA treated cell lines. Reduced relative OPN protein expression was observed in silenced primary tumor (WM278) and metastasis originated (WM1617) cell lines (*p* ≤ 0.05; Student’s t-test). All experiments were carried out at least three times, and the data are presented as the means (±SD). Black columns: untreated cells; gray columns: Negative Control siRNA (NC-siRNA) transfected cells; white columns: OPN-siRNA transfected cells.

We selected a primary and metastatic cell line pair (WM278–WM1617) that were originated from the same patient to determine the OPN-siRNA silencing efficiency, both cell lines had *BRAF* ^*V600E*^ mutation. Using validated OPN-specific siRNA, OPN expression was successfully inhibited in melanoma cell lines with high OPN expression. AllStars Negative Control siRNA (NC-siRNA) was used as control. OPN silencing was evaluated by qRT-PCR. siRNA-mediated OPN knockdown in melanoma cell lines resulted in a significant decrease in the OPN mRNA level (average inhibition rate of 60% compared to the negative control (*p* ≤ 0.05) (Figure 2A). Figure 2A clearly shows that OPN-specific siRNA successfully downregulated OPN gene expression in primary and metastatic melanoma cell lines. Simultaneously, the proliferation of the transfected cells decreased significantly when compared to the NC-siRNA treated and untreated control cells (Figure 2B). Similarly, decrease of OPN protein expression were observed (Figure 2C).

### Effect of RNAi on the Invasive Behaviour of Melanoma Cell

**FIGURE 3:**
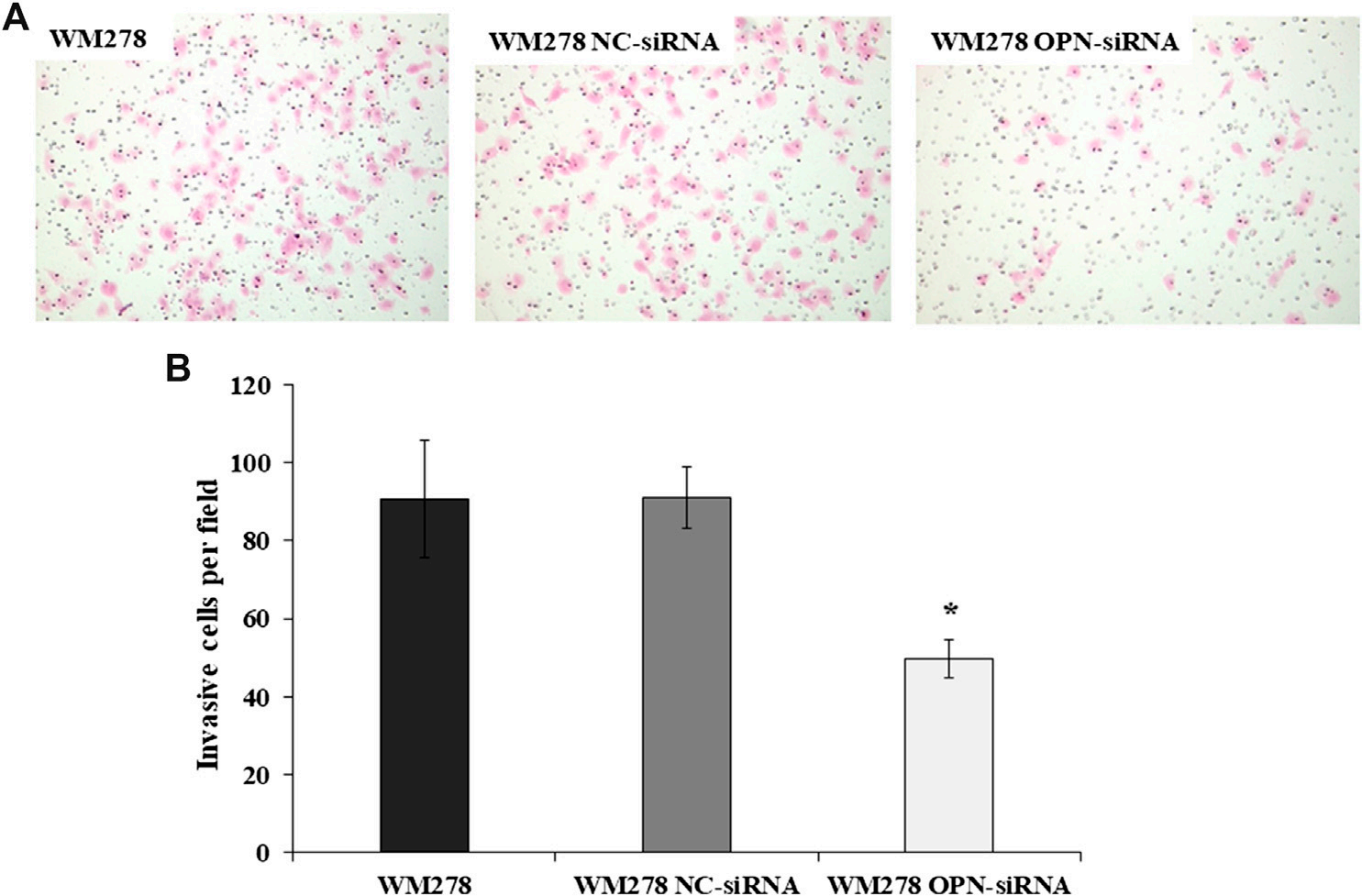
Invasive potential of WM278 cells. **(A)** Cells were cultured in Matrigel invasion chambers, fixed and stained with hematoxylin–eosin (×100 magnification). **(B)** The number of invaded cells are plotted for the untreated, NC-siRNA treated and OPN-siRNA silenced cells. The data are presented as the mean ± SD of three independent experiments. Invasion was significantly lower in the silenced cells compared to the control cell lines (untreated WM278 and NC-siRNA treated WM278) (*p* ≤ 0.05; Student’s t-test). Asterisk indicates significant difference between the control and OPN silenced cell lines. Black column: untreated cells; gray column: Negative Control siRNA (NC-siRNA) transfected cells; white column: OPN-siRNA transfected cells).

In order to define the invasive potential of OPN-siRNA silenced cells we evaluated the invasive potential of the cell lines after OPN knockdown. As an example, we show in Figure 3 that silencing of the OPN resulted in a significant decrease of invasive potential in the WM278 cells. On average, 91 (±16) control cells/field invaded onto the membrane surface, whereas only 49 (±10) OPN-siRNA transfected cells reached the filter, which is clearly demonstrated on the microscopic image (Figure 3A). The significantly fewer numbers of silenced cells on the membrane surface indicate that downregulated OPN expression is associated with decreased invasion (Figure 3B). We confirmed the reduced OPN expression by real-time qRT-PCR.

### Protein Expression Analysis of the Original and OPN siRNA-Transfected Cells

To determine protein expression differences between the original and OPN siRNA-transfected cell lines, we used a Proteome Profiler Human XL Oncology Array, which detects 84 cancer-related proteins.

**FIGURE 4:**
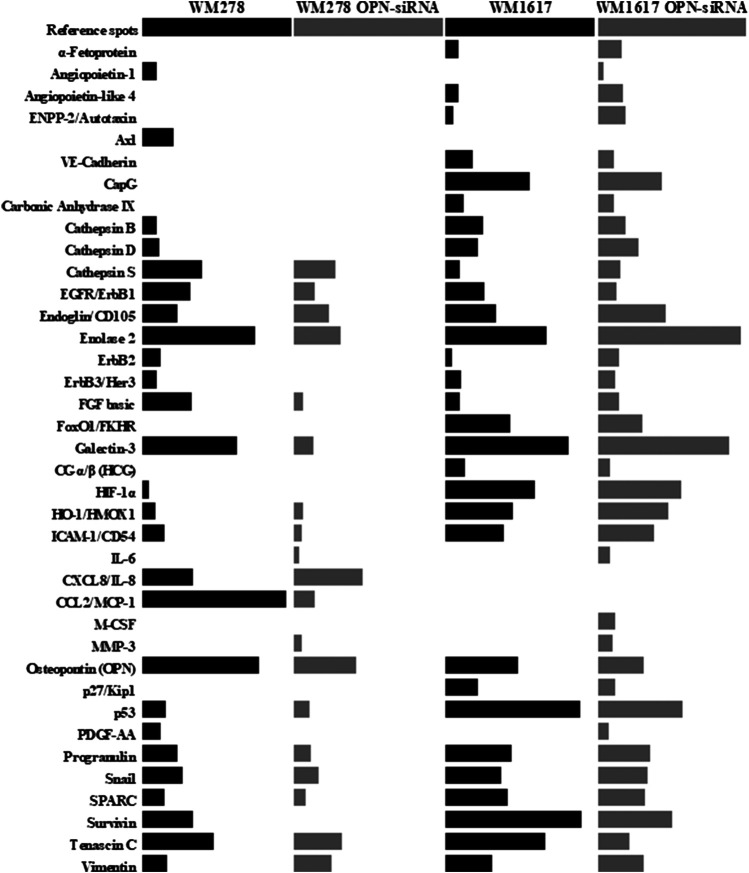
Protein expression profiles of controll (WM278 and WM1617) and OPN-siRNA transfected melanoma cell lines (WM278 OPN-siRNA and WM1617 OPN-siRNA). Protein expression was investigated using a Proteome Profiler Human XL Oncology Array Kit containing 84 cancer related genes. Only expressed proteins are displayed. The data are presented as the mean of three replicates/sample. The expression of proteins in the controll cells are labeled with black and the OPN-siRNA silenced cell lines are labeled with gray.

The name of the proteins, as well as the relative protein expressions of the melanoma cell lines are given in Supplementary Table S2. Out of the 84 proteins, 26 were expressed in the primary tumor originated WM278 and 32 proteins in the metastasis originated WM1617 cell lines. After OPN silencing only 19 proteins were expressed in the WM278 OPN-siRNA cell line, whereas 59 protein had detectable expression in the WM1617 OPN-siRNA cells. Comparision of the relative expression of proteins in the original (WM278 and WM1617) and OPN-siRNA silenced (WM278 OPN-siRNA and WM1617 OPN-siRNA) cell lines are shown in Figure 4.

**FIGURE 5:**
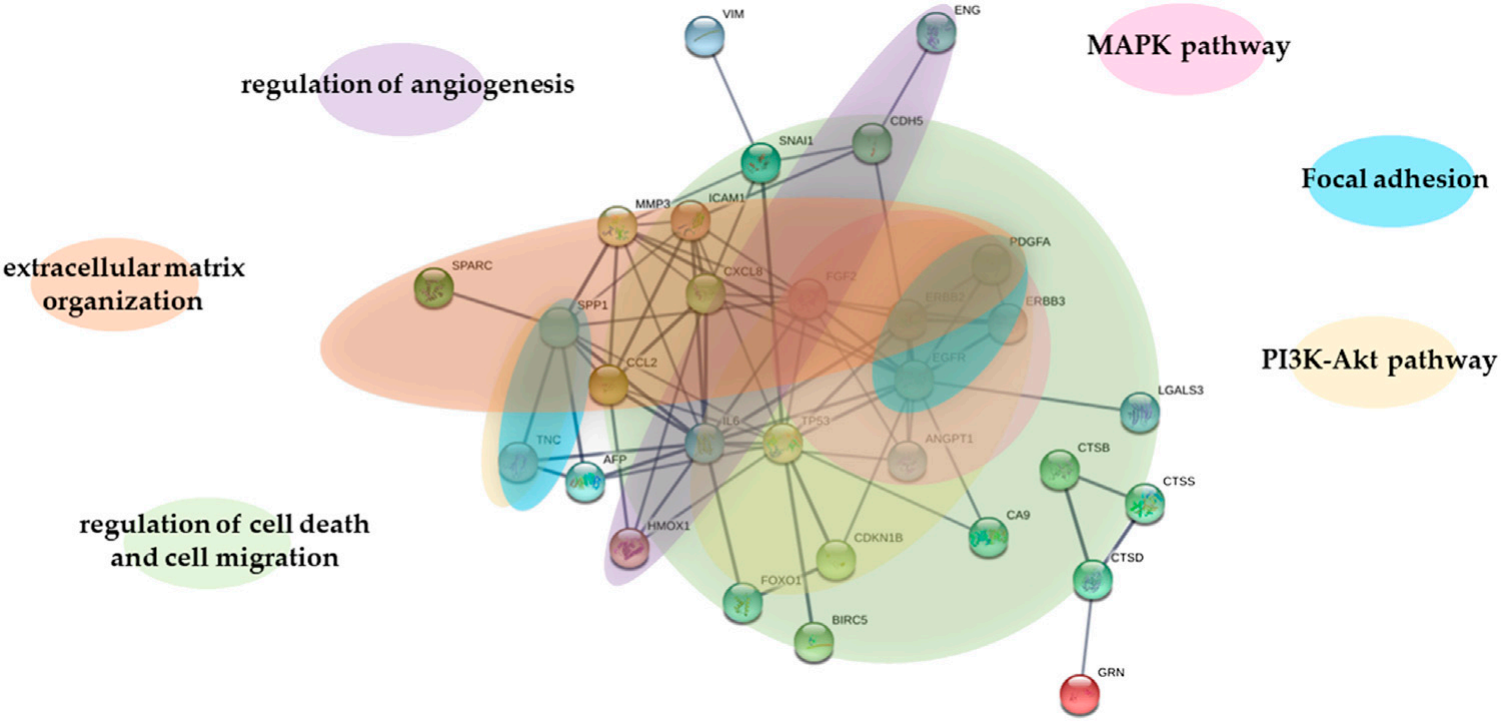
STRING analysis of proteins in WM278 and WM1617 cells. STRING database version 11.0 (http://string-db.org) was used to determine the protein-protein interactions of the 38 proteins differentially expressed in the original and transfected cells. Interactions predicted with high confidence level: 0.700 were included in the analyses, and proteins with no predicted interactions were removed. STRING: Search Tool for the Retrieval of Interacting Genes; AFP: alpha-fetoprotein; ANGPT1: angiopoietin-1; BIRC5: survivin; CA9: carbonic anhydrase IX; CCL2: C-C motif chemokine two; CDH5: VE-cadherin; CDKN1B: cyclin-dependent kinase inhibitor 1B/p27; CTSB: cathepsin B; CTSD: cathepsin D; CTSS: cathepsin S; CXCL8: interleukin-8; EGFR: epidermal growth factor receptor; ENG: endoglin; ERBB2: receptor tyrosine-protein kinase erbB-2; ERBB3: receptor tyrosine-protein kinase erbB-3; FGF2: basic fibroblast growth factor; FOX01: forkhead box protein O1; GRN: progranulin; HMOX1: heme oxygenase one; ICAM1: intercellular adhesion molecule one; IL-6: interleukin-6; LGALS3: galectin-3; MMP3: matrix metallo-proteinase-3; PDGFA: platelet-derived growth factor AA; SNAI1: snail; SPARC: secreted protein acidic and rich in cysteine; SPP1: osteopontin; TNC: tenascin C; TP53: cellular tumor antigen p53; VIM: vimentin; MAPK pathway: The Mitogen-Activated Protein Kinase Pathway; PI3K-Akt pathway: phosphatidylinositol 3-kinase protein kinase B signaling pathway.

To predict protein-protein interactions along with putative pathways we used STRING analysis for the differentially expressed proteins. STRING generated interconnected protein network with a high confidence level 0.700. As shown in Figure 5, original and transfected cells showed a differential protein interactions. Proteins formed multiple clusters, one is a cluster of extracellular matrix organization, the other is a cluster of regulation of angiogenezis, cell death and cell migration, and the final one is a cluster of PI3K-Akt, MAPK and focal adhesion signaling pathway.

**FIGURE 6:**
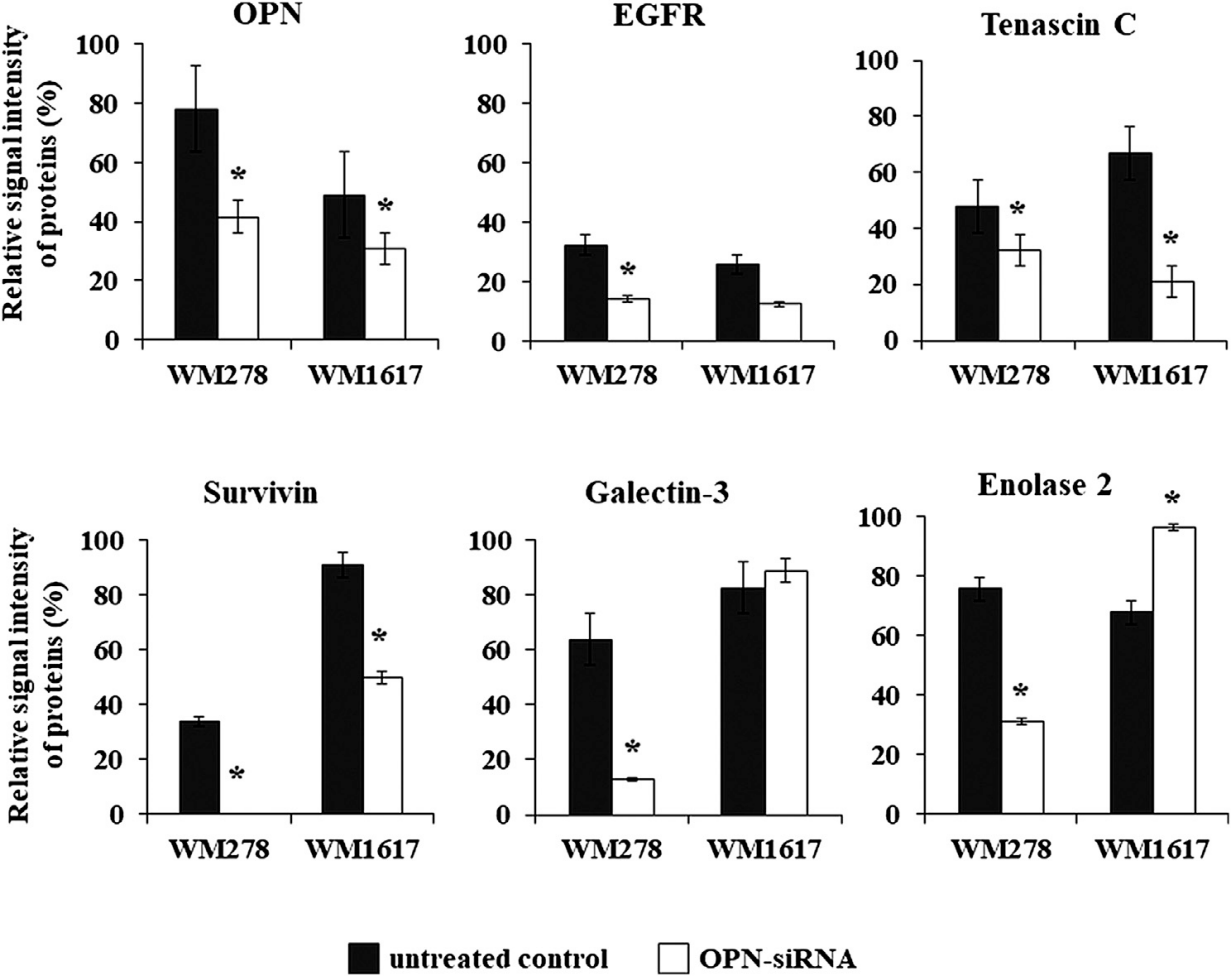
Protein expression of six proteins (OPN, EGFR tenascin C, enolase 2, galectin-3 and survivin) in the unterated (WM278 and WM1617) and in the OPN silenced melanoma cell lines (WM278 OPN-siRNA and WM1617 OPN-siRNA) (*p* ≤ 0.05; Student’s t-test). The data are presented as the mean ± SD of the relative signal intensity (three replicates/sample). Black columns: untreated cells; white columns; OPN-siRNA silenced cells. Asterisks represent significant difference.

Beside OPN, altered expression of five proteins were detected in both siRNA transfected cell lines. In addition to OPN, we found significantly decreased expression of EGFR, tenascin C and survivin (*p* ≤ 0.05). Galectin -3 and enolase two were significantly downregulated in the WM278 OPN-siRNAprimary tumor originated cell line (average inhibition rate 60%), in contrast, the expression of these two proteins were increased in the OPN-siRNA silenced metastasis originated WM1617 cells (Figure 6).

In order to validate the proteome profiler data we performed gene expression analysis. The gene expression pattern of the six genes (OPN, EGFR, tenascin C, survivin, galectin-3 and enolase 2) changed by the same direction as we have seen on the Proteome Profiler array (Supplementary Figure S3).

## Discussion

OPN is considered as one of the key molecules in the tumorigenesis, progression and metastatic dissemination of different malignancies, including melanoma [8, 31, 32]. Maier et al. determined OPN protein levels in the plasma of melanoma patients and found that OPN is a promising novel biomarker for the detection of metastatic tumors; these authors also concluded that the combination of plasma OPN levels with the well-established serological tumor marker S100 might enhance the prediction of melanoma metastasis [33]. Several investigations of different tumor types have documented that OPN expression is related to metastatic potential and is a useful diagnostic and therapeutic biomarker for different types of cancer [6, 8, 34]. Understanding the molecular mechanism of OPN expression during tumor progression can help to develop novel diagnostic and therapeutic approaches. The aim of our study was to determine the relative gene and protein expression levels of OPN in melanoma cell lines and melanoma tissues with different biological properties. Similar to previous studies, we found that OPN overexpression is closely associated with melanoma metastasis [8]. We successfully inhibited OPN expression in melanoma cell lines by siRNA and compared the invasive properties of the transfected cell lines to the original ones.

In the last decade, RNAi, which is a post-transcriptional mechanism for inhibiting gene expression, has shown promising results in molecular-targeting gene therapy for different types of cancer [35, 36]. OPN knockdown was shown to have antimetastatic and antitumorigenic effects in different cancers [37 – 40]; however, little data for malignant melanoma are available [12, 41]. In this study, we revealed that OPN expression can be downregulated using OPN-specific siRNA in primary and metastatic melanoma cell lines. We observed decrease in cell proliferation and cell migration after effectively silencing the OPN gene. Because silencing a gene can induce changes in the expression of different proteins, we used Proteome Profiler Human XL Oncology Array to define the expression levels of 84 cancer-related proteins. Comparing the expression patterns of the original and transfected cell lines, we detected a number of differentially expressed proteins. The OPN protein expression levels decreased in both OPN-silenced cell lines (primary tumor-and metastasis-derived). Altered expression of other proteins included EGFR, tenascin C, survivin, galectin-3 and enolase 2. Marked reductions in the expression of OPN and the antiapoptotic protein survivin were detected in association with BRAF inhibitor resistance by us and others [42]. Survivin is an apoptosis inhibitor, and its expression is associated with poor prognosis in cancers [43]. Recently, Chen and co-workers described that survivin has an important role in tumorigenesis [44]. Decreased tenascin C and survivin protein expression in the OPN-siRNA transfected cell lines are in very good agreement with the OPN expression levels, which play important roles in various metastasis-associated mechanisms, including cell proliferation, apoptosis, invasion and migration [45]. The expression of tenascin C has been shown to be essential in cellular invasion and migration and is important during the development of metastasis [46]. According to recent studies, there is a significant association between OPN and EGFR expression in clear cell renal cell carcinoma [47]. Similar to our findings, OPN inhibition led to decreased EGFR expression and increased apoptotic cell death. Apoptosis was significantly enhanced in OPN knockout mice and was accompanied by EGFR downregulation [48]. Furthermore, we found that decreased OPN expression was associated with lower galectin-3 and enolase two protein levels in the primary tumor-derived transfected cell line; however, the expression of these proteins was increased in the metastatic cell line after OPN silencing. Galectin-3 expression is a marker and promoter of progression and metastasis in many tumors [49, 50] interestingly, galectin-3 and OPN were proposed as potential targets (or at least predictors) in future personalized antiaging therapies [51]. Galectin-3 and enolase two overexpression were detected in the transfected metastatic cell line, and we assume that both play an important role in promoting the aggressive phenotype of melanoma cells. The simultaneous expression of these proteins and their role in tumor progression have not been previously described in melanoma. Li et al. described a significant increase in the expression levels of galectin-3 and enolase 2 (and other proteins) that associated with hepatocellular carcinoma progression [52]. Enolase two is a specific molecular marker that is used in cancer diagnosis and can promote the migration and invasion of tumor cells by remodeling the actin cytoskeleton [53].

Based on the already published data, we assume that the proteins altered in the OPN-siRNA silenced cells are involved in the following biological processes: extracellular matrix binding (galectin-3 and OPN) and protein dimerization (EGFR, galectin-3, enolase two and survivin) [7, 54 – 56]. It was shown that down-regulation of galectin-3 and the other proteins were associated with decreased migration, invasion and reduced tumor growth [55, 57]. Nevertheless, galectin-3 has a regulatory role in cancer stemness related pathways beside other pathways the EGFR/FGFR pathway is also involved and it was published that OPN induced migration and invasion is strongly associated with activation of different EGF receptors [58]. Consequently, decreased level of OPN and other proteins might contribute to the less aggressive phenotype. Additionally, three of the altered proteins (EGFR, OPN and tenascin C) are related to the PI3K-Akt signaling pathway. On the other hand EGFR, galectin-3 and OPN potentially influence the extracellular signal-regulated RAF/MEK/ERK pathway [59 – 62], and both pathways are fundamental in melanoma tumorigenesis [63, 64]. EGFR, OPN and tenascin C have crucial role in the focal adhesion pathway, the multiprotein focal adhesion complexes silenced by OPN-siRNA can promote the connection between the extracellular matrix and cytoskeleton, and functionally control cell proliferation, differentiation, and motility [65]. Alterations of these pathways are significantly important in the pathogenesis of melanoma by affecting tumorigenesis, cellular growth, chemoresistance, invasion and migration [1, 66].

In summary, we showed that a high expression level of OPN is associated with a more aggressive phenotype in melanoma. Our data clearly showed protein expression differences between the OPN-siRNA-transfected and the untreated melanoma cell lines. These results revealed for the first time that silencing the OPN gene influence proliferation and invasion of melanoma cells by effecting EGFR, tenascin C, survivin, galectin-3 and enolase two expression. Our data suggest that OPN might be an ideal target for drug development and therapies.

## Data Availability Statement

The raw data supporting the conclusions of this article will be made available by the authors, without undue reservation.

## Ethics Statement

The studies involving human participants were reviewed and approved by Regional and Institutional Ethics Committee of the University of Debrecen, Hungary (DE RKEB/IKEB: 4820-2017). The patients/participants provided their written informed consent to participate in this study.

## Author Contributions

Conceptualization, MB; Data curation, TK, KJ, VK, IS, HB, and AM; Formal analysis, TK, KJ, and IS; Funding acquisition, RÁ and MB; Investigation, TK, VK, RÁ, and MB; Methodology, TK, KJ, VK, IS, HB, and AM; Supervision, MB; Validation, TK; Writing–original draft, TK and VK; Writing–review and editing, RÁ and MB.

## Funding

This research was co-financed by the National Research Development and Innovation Fund (grant number K-112327), by the European Regional Development Fund (GINOP-2.3.2-15-2016-00005), by the ÚNKP-19-3 New National Excellence Program of the Ministry for Innovation and Technology as well as by the Hungarian Academy of Sciences (MTA11010 and TK2016-78).

## Conflict of Interest

The authors declare that the research was conducted in the absence of any commercial or financial relationships that could be construed as a potential conflict of interest.
