# Supplementary figures and images for: Silencing Osteopontin Expression Inhibits Proliferation, Invasion and Induce Altered Protein Expression in Melanoma Cells
Source: Pathol Oncol Res. 2021 Mar 5;27:581395. doi: 10.3389/pore.2021.581395 (PMC8262222; doi:10.3389/pore.2021.581395)

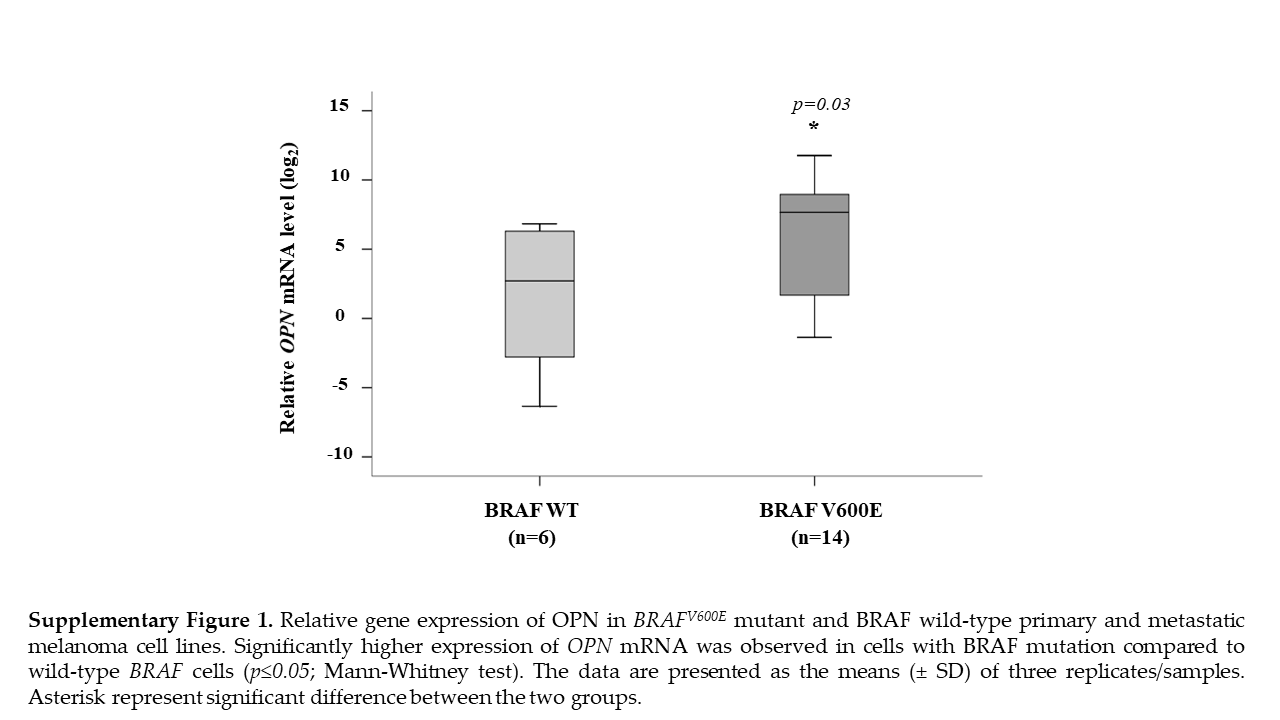

Supplement: Supplementary file 1 [file Image1.TIF]
