# Supplementary material for: Silencing Osteopontin Expression Inhibits Proliferation, Invasion and Induce Altered Protein Expression in Melanoma Cells
Source: Pathol Oncol Res. 2021 Mar 5;27:581395. doi: 10.3389/pore.2021.581395 (PMC8262222; doi:10.3389/pore.2021.581395)
Supplement: Supplementary file 2 [file TableS1.pdf]

Supplementary Table 1. Clinical–pathological data of primary and metastatic melanoma tissue samples used in qRT-PCR analysis

Sample number	Sex	Age at initial diagnosis (year)	Histological subtype	Breslow thickness (mm)	Clark stage	TNM status
Primary melanoma samples						
P04	F	40	SSM	7.00	IV	T4bN0M0
P05	M	52	SSM	3.20	IV	T3bN0M0
P06	M	74	SSM	5.00	III-IV	T4bN0M0
P08	M	41	SSM	14.0	V	T4b2bM3
P09	F	54	SSM	11.0	n.d.	T4N2
P10	M	71	SSM	0.15	III	T1aNxMx
P12	M	68	SSM	3.50	III-IV	T3bN0M0
P13	M	51	SSM	2.42	III	T3aNxMx
P14	F	62	SSM	0.57	III	T1aN0M0
P15	M	53	SSM	0.72	III	T1aN0M0
P16	F	72	SSM	0.24	III	T1aN0M0
P18	M	51	SSM	1.33	III	T2aN0M0
P19	F	54	SSM	0.65	III	T1aN0M0
P23	F	70	SSM	0.36	III	T1aN0M0
P24	F	70	SSM	0.24	III	T1aN0M0
P25	F	44	SSM	0.24	III	T1aN0M0
P26	M	47	SSM	3.14	III	T3aN0M0
P29	F	68	SSM	1.05	IV	T2aN0
P30	M	64	SSM	0.30	II	T1aN0
P31	F	75	SSM	3.00	IV	T3bN0
P32	M	73	SSM	2.80	IV	T2aNx
P34	M	51	SSM	3.20	IV	pT3a
P35	F	86	SSM	2.40	IV	pT3bN0
P36	F	72	SSM	2.40	IV	pT3a
P01	F	71	NM	7.00	IV	T4bN0M0
P02	M	38	NM	12.00	IV	T4bN0M0
P03	F	77	NM	12.00	V	T4bN3M0
P07	M	44	NM	25.00	V	T4bN0M0
P11	M	63	NM	8.00	V	T4bN0M0
P17	F	61	NM	2.64	III	T3aN0M0
P22	F	44	NM	3.72	III	T3aN0M0
P27	M	72	NM	3.60	III	T3bNxMx
P28	F	83	NM	3.50	IV	T3b
P33	M	77	NM	2.20	IV	pT3bN0

Sample number	Sex	Age at initial diagnosis (year)	Localization of metastasis
Melanoma metastasis			
M01	M	n.d.	Distant
M02	n.d.	n.d.	Distant
M03	M	81	rSC/C
M04	M	75	rLN
M05	F	74	rSC/C
M06	F	30	rLN
M07	M	72	rLN
M08	n.d.	n.d.	Distant
M09	M	65	rLN
M10	M	52	rSC/C
M11	M	70	rSC/C
M12	M	81	Distant

F: female; M: male; n.d.: no data available; SSM: superficial spreading melanoma; NM: nodular melanoma; rLN: regional lymph node; rSC/C: regional (sub)cutaneous.
