# Supplementary material for: Silencing Osteopontin Expression Inhibits Proliferation, Invasion and Induce Altered Protein Expression in Melanoma Cells
Source: Pathol Oncol Res. 2021 Mar 5;27:581395. doi: 10.3389/pore.2021.581395 (PMC8262222; doi:10.3389/pore.2021.581395)
Supplement: Supplementary file 3 [file TableS2.pdf]

**Supplementary Table 2. Relative protein expression of 84 cancer-related proteins in melanoma cell lines**

| Sl. No. | Positions of the Proteins on the Array | Proteins Name | WM278 | WM278 OPN-siRNA | WM1617 | WM1617 OPN-siRNA |
|---|---|---|---|---|---|---|
| | A1/A2 | **Reference spots** | 100 | 100 | 100 | 100 |
| 1 | A3/A4 | **$\alpha$-Fetoprotein** | 0 | 0 | 8.70 | 15.86 |
| 2 | A5/A6 | **Amphiregulin** | 0 | 0 | 0 | 4.18 |
| 3 | A7/A8 | **Angiopoietin-1** | 9.98 | 0 | 0 | 4.07 |
| 4 | A9/A10 | **Angiopoietin-like 4** | 0 | 0 | 8.20 | 17.06 |
| 5 | A11/A12 | **ENPP-2/Autotaxin** | 0 | 0 | 5.14 | 18.91 |
| 6 | A13/A14 | **Axl** | 21.18 | 0 | 0 | 0 |
| 7 | A15/A16 | **BCL-x** | 0 | 0 | 6.21 | 0 |
| 8 | A17/A18 | **CA125/MUC16** | 0 | 0 | 0 | 0 |
| 9 | A19/A20 | **E-Cadherin** | 0 | 0 | 0 | 0 |
| 10 | A21/A22 | **VE-Cadherin** | 0 | 0 | 18.57 | 10.46 |
| 11 | B3/B4 | **CapG** | 0 | 0 | 56.15 | 43.29 |
| 12 | B5/B6 | **Carbonic Anhydrase IX** | 0 | 0 | 11.97 | 10.52 |
| 13 | B7/B8 | **Cathepsin B** | 9.76 | 0 | 25.34 | 18.23 |
| 14 | B9/B10 | **Cathepsin D** | 11.03 | 0 | 21.97 | 27.24 |
| 15 | B11/B12 | **Cathepsin S** | 40.39 | 27.27 | 9.4 | 14.65 |
| 16 | B13/B14 | **CEACAM-5** | 0 | 0 | 0 | 6.1 |
| 17 | B15/B16 | **Decorin** | 0 | 0 | 0 | 5.24 |
| 18 | B17/B18 | **Dkk-1** | 0 | 0 | 0 | 0 |
| 19 | B19/B20 | **DLL1** | 0 | 0 | 4.83 | 9.58 |
| 20 | B21/B22 | **EGFR/ErbB1** | 32.34 | 14.19 | 25.8 | 12.42 |
| 21 | C3/C4 | **Endoglin/CD105** | 22.89 | 23.22 | 33.53 | 45.6 |
| 22 | C5/C6 | **Endostatin** | 0 | 0 | 0 | 0 |
| 23 | C7/C8 | **Enolase 2** | 75.83 | 31.06 | 67.93 | 96.61 |
| 24 | C9/C10 | **eNOS** | 0 | 0 | 0 | 4.37 |
| 25 | C11/C12 | **EpCAM/TROP1** | 0 | 0 | 0 | 3.12 |
| 26 | C13/C14 | **ER$\alpha$/NR3A1** | 0 | 0 | 0 | 0 |
| 27 | C15/C16 | **ErbB2** | 12.12 | 0 | 4.36 | 14.4 |
| 28 | C17/C18 | **ErbB3/Her3** | 10.06 | 0 | 9.82 | 11.01 |
| 29 | C19/C20 | **ErbB4** | 0 | 0 | 0 | 4.96 |
| 30 | C21/C22 | **FGF basic** | 33.02 | 6.36 | 8.89 | 13.77 |
| 31 | D1/D2 | **FoxC2** | 0 | 0 | 0 | 0 |
| 32 | D3/D4 | **FoxO1/FKHR** | 0 | 0 | 43.33 | 30.05 |
| 33 | D5/D6 | **Galectin-3** | 63.83 | 12.9 | 82.67 | 88.84 |
| 34 | D7/D8 | **GM-CSF** | 0 | 0 | 0 | 3.55 |
| 35 | D9/D10 | **CG $\alpha/\beta$ (HCG)** | 0 | 0 | 13 | 8.8 |
| 36 | D11/D12 | **HGF R/c-Met** | 0 | 0 | 0 | 0 |
| 37 | D13/D14 | **HIF-1$\alpha$** | 4.20 | 0 | 59.92 | 56.06 |
| 38 | D15/D16 | **HNF-3$\beta$** | | 0 | 0 | 4.63 |
| 39 | D17/D18 | **HO-1/HMOX1** | 9.15 | 5.51 | 45.2 | 47.36 |
| 40 | D19/D20 | **ICAM-1/CD54** | 14.56 | 4.89 | 38.85 | 37.7 |
| 41 | D21/D22 | **IL-2 R$\alpha$** | 0 | 0 | 0 | 0 |
| 42 | D23/D24 | **IL-6** | 0 | 3.62 | 0 | 8.23 |
| 43 | E1/E2 | **CXCL8/IL-8** | 34.09 | 46.58 | 0 | 0 |
| 44 | E3/E4 | **IL-18 BPa** | 0 | 0 | 0 | 4.18 |
| 45 | E5/E6 | **Kallikrein 3/PSA** | 0 | 0 | 0 | 4.81 |
| 46 | E7/E8 | **Kallikrein 5** | 0 | 0 | 0 | 0 |
| 47 | E9/E10 | **Kallikrien 6** | 0 | 0 | 0 | 0 |
| 48 | E11/E12 | **Leptin** | 0 | 0 | 0 | 0 |
| 49 | E13/E14 | **Lumican** | 0 | 0 | 0 | 0 |

| Sl. No. | Positions of the Proteins on the Array | Proteins Name | WM278 | WM278 OPN-siRNA | WM1617 | WM1617 OPN-siRNA |
|---|---|---|---|---|---|---|
| 50 | E15/E16 | **CCL2/MCP-1** | 96.97 | 14.32 | 0 | 0 |
| 51 | E17/E18 | **CCL8/MCP-2** | 0 | 0 | 0 | 5.03 |
| 52 | E19/E20 | **CCL7/MCP-3** | 0 | 0 | 0 | 0 |
| 53 | E21/E22 | **M-CSF** | 0 | 0 | 0 | 11.83 |
| 54 | E23/E24 | **Mesothelin** | 0 | 0 | 0 | 9.56 |
| 55 | F1/F2 | **CCL3/MIP-1α** | 0 | 0 | 0 | 0 |
| 56 | F3/F4 | **CCL20/MIP-3α** | 0 | 0 | 0 | 3.63 |
| 57 | F5/F6 | **MMP-2** | 0 | 0 | 0 | 3.77 |
| 58 | F7/F8 | **MMP-3** | 0 | 5.13 | 0 | 10.13 |
| 59 | F9/F10 | **MMP-9** | 0 | 0 | 0 | 0 |
| 60 | F11/F12 | **MSP/MST1** | 0 | 0 | 0 | 4.06 |
| 61 | F13/F14 | **MUC-1** | 0 | 0 | 0 | 4.26 |
| 62 | F15/F16 | **Nectin-4** | 0 | 0 | 0 | 4.07 |
| 63 | F17/F18 | **Osteopontin (OPN)** | 78.09 | 41.58 | 48.9 | 30.72 |
| 64 | F19/F20 | **p27/Kip1** | 0 | 0 | 22.07 | 12.24 |
| 65 | F21/F22 | **p53** | 15.15 | 10.87 | 89.85 | 57.03 |
| 66 | F23/F24 | **PDGF-AA** | 12.67 | 0 | 0 | 6.78 |
| 67 | G1/G2 | **CD31/PECAM-1** | 0 | 0 | 0 | 0 |
| 68 | G3/G4 | **Progesterone R/NR3C3** | 0 | 0 | 0 | 0 |
| 69 | G5/G6 | **Progranulin** | 23.81 | 11.17 | 44.49 | 34.95 |
| 70 | G7/G8 | **Prolactin** | 0 | 0 | 0 | 7.37 |
| 71 | G9/G10 | **Prostasin/Prss8** | 0 | 0 | 0 | 0 |
| 72 | G11/G12 | **E-Selectin/CD62E** | 0 | 0 | 0 | 0 |
| 73 | G13/G14 | **Serpin B5/Maspin** | 0 | 0 | 0 | 0 |
| 74 | G15/G16 | **Serpin E1/PAI-1** | 0 | 0 | 0 | 5.94 |
| 75 | G17/G18 | **Snail** | 27.51 | 16.16 | 37.31 | 33.77 |
| 76 | G19/G20 | **SPARC** | 15.04 | 7.98 | 41.63 | 31.95 |
| 77 | G21/G22 | **Survivin** | 33.94 | 0 | 90.78 | 49.76 |
| 78 | G23/G24 | **Tenascin C** | 47.84 | 32.16 | 66.78 | 21.15 |
| 79 | H1/H2 | **Thrombospondin-1** | 0 | 0 | 0 | 0 |
| 80 | H3/H4 | **Tie-2** | 0 | 0 | 0 | 5.18 |
| 81 | H5/H6 | **u-Plasminogen Activator/Urokinase** | 0 | 0 | 0 | 4.4 |
| 82 | H7/H8 | **VCAM-1/CD106** | 0 | 0 | 0 | 3.7 |
| 83 | H9/H10 | **VEGF** | 0 | 0 | 0 | 2.77 |
| 84 | H11/H12 | **Vimentin** | 16.77 | 25.68 | 31.25 | 31.06 |
